# Early Detection of COVID-19 Waves From Cases in a Neighboring Country With an Open Border

**DOI:** 10.3389/fpubh.2021.739738

**Published:** 2021-10-29

**Authors:** Anil Kamat, Amrita Sah

**Affiliations:** ^1^Department of Mechanical, Aerospace and Nuclear Engineering, Rensselaer Polytechnic Institute, Troy, NY, United States; ^2^Nepalgunj Medical College, Nepalgunj, Nepal

**Keywords:** COVID-19, open border, wave detection, border closure, COVID-19 cases correlation

## Abstract

Border closure or travel restriction is a critical issue as closing the border early can badly affect the economy of the country, whereas substantial delay can put human lives at stake. While many papers discuss closing the border early in the pandemic, the question of when to close the border has not been addressed well. We have tried to estimate a date of closing the border by taking the reference of a neighboring country with a high correlation in Covid-19 incidence. Here we have used non-linear methods to probe the landscape of correlation between temporal COVID-19 incidences and deaths. We have tested our method on two neighboring countries, Nepal and India, with open borders, where closing the borders are among the top priorities to reduce the spread and spill-out of variants. We have selected these countries as they have close connectivity and intertwined socio-economic network with thousands of people crossing the border every day. We found the distance correlation for COVID-19 incidence between these countries to be statistically significant (*p* < 0.001) and there is a lag of 6 days for maximum correlation. In addition, we analyzed the correlation for each wave and found the distance correlation for the first phase is 0.8145 (*p* < 0.001) with a lag of 2 days, and the distance correlation for the second wave is 0.9685 (*p* < 0.001) without any lag. This study can be a critical planning tool for policymakers and public health practitioners to make an informed decision on border closure in the early days as it is critically associated with the legal and diplomatic agreements and regulations between two countries.

## Introduction

By the time World Health Organization (WHO) declared respiratory infectious disease (COVID-19) due to a novel coronavirus (SARS-CoV-2) as a pandemic (1) on March 11, 2020, the virus had already crossed the border of China and had spread into 114 countries due to its high reproduction number compared to other SARS coronaviruses (2). The globalization and ease of mobility from one country to another have highly contributed to the rapid and far spread of COVID-19 around the world (3). Greater connectedness and integration among countries naturally increase pathways for pathogens to cross borders and emerge in local populations. High-frequency trade and international travel facilitate global disease transmission while open borders and social networks have rendered neighboring countries more vulnerable to the emergence and spread of infectious diseases (4–6). When people from highly affected territories move to low affected territories, it puts more pressure and challenge on the local public health system. It is well-established that a country with a low socioeconomic position suffers more (7). On the other hand, greater economic globalization leads to slower adaptation of policies due to legal bindings on travel and trade agreements (8). Countries with an open border and people working abroad may be less inclined to closing the border in order to minimize economic losses. Closing the border with some delay/lag and slowly reducing mobility is a common feature of most countries (9). In contrast, countries like China, Japan, and Korea which adopted stringent early travel restrictions, were successful in containing the virus whereas the countries with a porous border like Nepal and India faced worse conditions.

The relationship between Nepal and India is one of the oldest diplomatic relations in the world. These two countries are among the handful of countries that share open borders. People of either country can easily get into the other country without requiring any official documentation (10). Similar religions and social norms have created intense social-economic networks across the open border. The open border has greatly helped people of both countries to do trade and business which has created employment opportunities for the locals (11). Nevertheless, these benefits come with some risks, as the local people also share the potential vulnerability to disease transmission and outbreak. In fact, risk due to population outflow is higher than any other factors like geographical proximity and similarity in economic conditions. The open border and lack of documentation make it even harder to trace (12) and constrain the outbreak within one country. Thus, it is essential not only to know the strength of the relationship of COVID-19 incidence between these two countries but also to know the lead or the lag in the days for maximum similarity of the new cases.

A simple yet effective method to measure the relationship between the incidences is the correlation analysis. Depending upon the stationarity of the time series, the correlation method is selected to produce a meaningful relationship. As the traditional Pearson correlation can give a spurious relationship if the time series is not stationary, Distance Correlation is usually preferred to measure the dependencies of two time-series. Distance correlation is a new method to quantify the strength of dependences between two random vectors of arbitrary dimension (13). Distance correlation is the exact equivalence of Brownian correlation (14). Unlike the traditional Pearson product-moment correlation method, the distance correlation can measure the linear as well as the non-linear relationship between two variables (15). Further, 0 in distance correlation means absolute independencies, i.e., the distance correlation between two variables is zero if and only if they are statistically independent. The Distance Correlation has already been used for measuring dependencies in power-law growth of four countries (16). To the best of our knowledge, this is the first time anyone has analyzed the relationship between the COVID-19 incidence rate in Nepal and India to find out the number of days, it takes the effect to propagate from one country to another using cross-correlation. Given the complexity of pandemic decisions, governments face a dilemma about the beginning of travel restriction, and our analysis provides the lag in days, which may help the policymakers of either country to prepare intervention measures and plan strategies to control transmission ahead of the pandemic in future to save thousands of lives.

## Materials and Methods

### Data

All the data used in the study is taken from the Data Repository by the Center for Systems Science and Engineering (CSSE) at Johns Hopkins University (17). Individual-level data from the open-access datasets of COVID-19 cases for Nepal and India was considered from January 25, 2020 to May 18, 2021, and January 30, 2020, to May 18, 2021, respectively. All the data from the recorded first case till the date were selected for this study. The beginning dates of data are few months before the global pandemic declaration on March 11. The data from Nepal were reported by the ministry of health and population and the data from India were reported by the ministry of health and family welfare. All the analysis of data was carried out in Matlab® (18) (R2019b, http://www.mathworks.com). The Matlab® source code and the dataset used for this analysis can be found in the following GitHub repository: https://github.com/anilkamat/Covid-19_NepIndCorrelation.

### Wave Detection

To detect the onset and end of the first and second waves of coronavirus, we have used the multiple change-point detection (CPD) method (19). The CPD method depends on the statistical properties of the time series. A change point is a sample or time instant at which some statistical property of a time series changes abruptly. We considered a change if the mean and slope of the time series have changed mostly abruptly. The approach of this method is to minimize a cost function over possible numbers and locations of change points. The whole time series is divided into *n*+1 segments for the detection of *n* change points and the empirical property is estimated for each of the sections. Then, at each point, the deviation of the statistical property from the empirical estimates is computed. Similarly, the deviation of each section is calculated and added together to find the total residual error. The residual error for each of the division points along the time series is computed and compared to find the point with minimum residual error. Consider a time-series as:


$$\begin{array}{l} y_{1:n}=\left(y_{1},y_{2}\ldots ..y_{n}\right) \end{array}$$

The model can have *m* number of change points which will split the series into *m*+1 segments:


$$\begin{array}{l} t_{1:m}=\left(t_{1},\;\ldots \ldots \;t_{m}\right) \end{array}$$

Each change point position is an integer between 1 and *n–*1 inclusive. Here, we have used the linear fit statistical property which uses the total deviation of the sum of squared differences between the time-series values and the predictions of the least-squares linear fit through the values of the line segment. The best line through *y*_*m*_, *y*_*m*+1_…..*y*_*n*_ is given by:


$$\begin{array}{l} ŷ=\;\frac{S_{xt}{|}_{m}^{n}}{S_{tt}{|}_{m}^{n}}\left(t-mean\left(\left[t_{m}\ldots \ldots \ldots t_{n}\right]\right)\right)\\ \;+mean\left(y_{m},y_{m+1}\ldots ..y_{n}\right) \end{array}$$

Where the sum of square *S*_*xy*_ is:


$$\begin{array}{l} S_{xt}{|}_{m}^{n}=\;\overset{n}{\underset{r=m}{\sum }}\left(x-mean\left(\left[x_{m}\ldots \ldots \ldots x_{n}\right]\right)\right)\\ \;\left(y-mean\left(\left[y_{m}\ldots \ldots \ldots y_{n}\right]\right)\right) \end{array}$$

And the error sum of square (SSE):


$$\begin{array}{l} SSE=\overset{n}{\underset{i=m}{\sum }}{\left(x_{i}-\hat{x}\left(t_{i}\right)\right)}^{2}=\;S_{xx}{|}_{m}^{n}-\;\frac{{S_{xt}}^{2}{|}_{m}^{n}}{S_{tt}{|}_{m}^{n}}\\ \;=\left(n-m+1\right)var\left(\left[x_{m}\ldots x_{n}\right]\right)\\ \;-\frac{{\left(\overset{n}{\underset{i=m}{\sum }}\left(x_{i}-mean\left(\left[x_{m}\ldots \;x_{n}\right]\right)\right)\left(i-mean\left(\left[m\;m+1\ldots n\;\right]\right)\right)\right)}^{2}}{\left(n-m+1\right)var\left(\left[m\;m+1\ldots n\right]\right)} \end{array}$$

If there are *K* change points the function minimizes:


$$\begin{array}{l} J\left(K\right)=\;\overset{k-1}{\underset{0}{\sum }}\overset{k_{r+1}-1}{\underset{i=\;k_{r}}{\sum }}△\left(x_{i};\chi \left(\left[x_{k_{r}}\ldots \ldots \;x_{k_{r+1}}-1\right]\right)\right)+\beta K \end{array}$$

Where β is the proportionality constant which penalizes the function to minimize the residual error.

### Dependency Analysis

We first tested the stationarity of the data using Dickey-Fuller's test (20) to see if the time-series contains a unit root and found the time-series were not stationary, which requires non-linear correlation methods like distance correlation. Distance measure takes the value of [0, 1], where the 0, implies absolute independence and 1 implies the perfect correlation between the two vectors. It is free of matrix inversion and estimation of parameters and can be computed as below.


$$\begin{array}{l} Cor_{D}\left(x,y\right)=\;\frac{Cov_{D}\left(x,y\right)}{\sigma_{D}\left(x\right)\sigma_{D}\left(y\right)} \end{array}$$

Where, *Cov*_*D*_(*x, y*) is the distance covariance between two variables and σ_*D*_ is the standard deviation.

Statistical significance was defined where *p*-values were < 0.01. Note: two countries can be considered to be dependent if the correlation of the confirmed new cases between them is >0.5 (21). To find the lag for optimal similarity of the cases, we have used cross-correlation (22). The value of the lag with the highest correlation coefficient represents the best fit between the two series.

## Results

**Figure 1:**
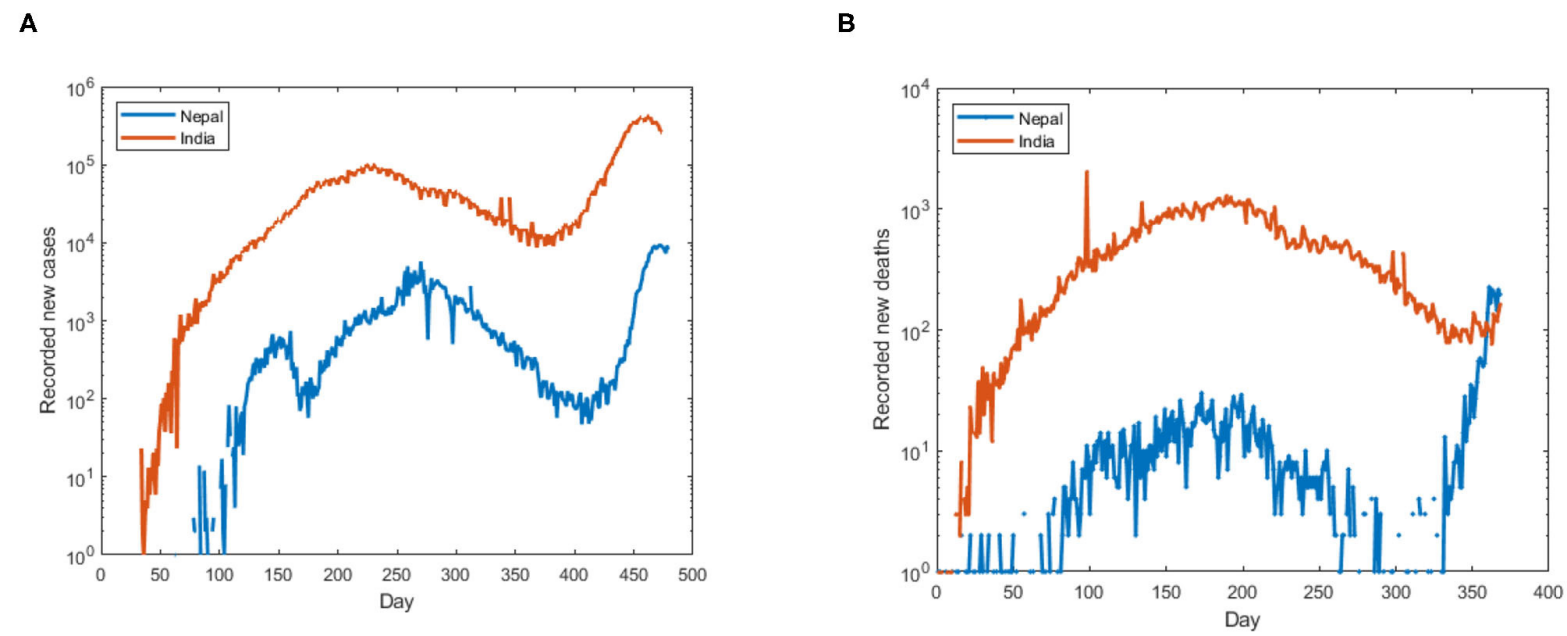
**(A)** Recorded new cases of COVID-19 in Nepal and India, **(B)** recorded new deaths of COVID-19 in Nepal and India. The *y*-axis is in log scale to make the cases visible.

**Figure 2:**
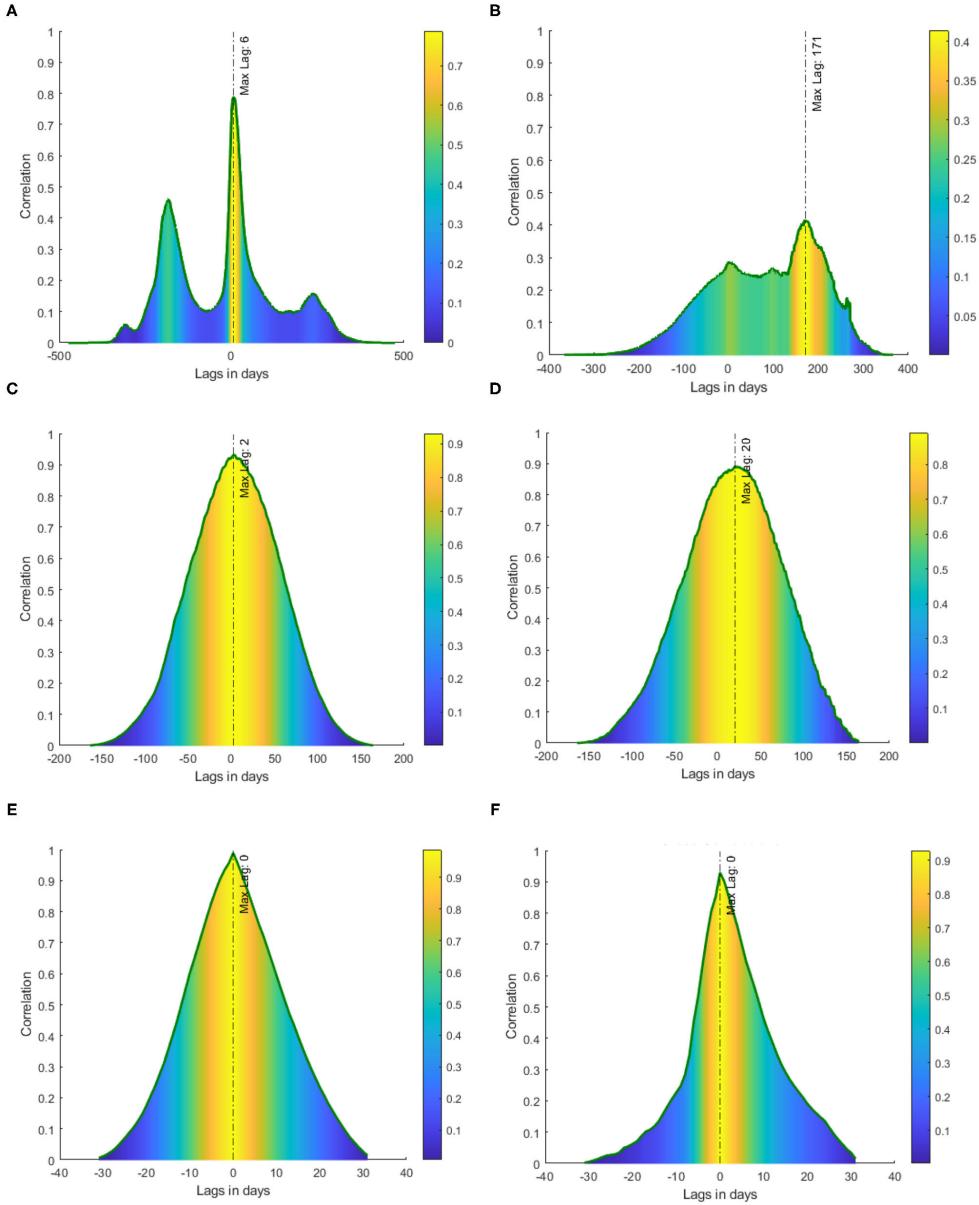
Cross-correlation between Nepal and India with lags in days. The color represents the magnitude of cross-correlation at each lag. **(A)** Cross-correlation of incident cases, **(B)** cross-correlation of new deaths, **(C)** cross-correlation of incident cases in first wave, **(D)** cross-correlation of daily deaths in the first wave, **(E)** cross-correlation of incident cases in the second wave, and **(F)** cross-correlation of daily deaths in the second wave.

We first tested the presence of unit root in the data by employing Dickey-Fuller's test of stationarity. We found the time-series of incidence rate and mortality rate of Nepal and India (Figures 1A,B) were non-stationary (*p* > 0.05), the presence of unit root suggests the use of distance correlation. Thus, to quantify the non-linear dependency, we used distance correlation, and a significant distance correlation coefficient of 0.6337 (*p* < 0.001, *N* = 474) was observed between the daily incidence of Nepal and India. We then performed cross-correlation for different lags of days which provides the difference in days for a maximum fit of both the incidence rates. Thus, to report the dependencies of incidence rate at each time lag, the normalized cross-correlation coefficients were calculated for a forward and backward lag of 500 days. We can observe (see Figure 2A) two peaks in the cross-correlation, first, a correlation of 0.4602 occurs at the lag of 183 days, and the second of 0.78848 occurs at the lag of 6 days. Similarly, we found the distance correlation for the daily new deaths (see Figure 2B) to be non-linearly related with a correlation coefficient of 0.4702 (*p* < 0.001, *N* = 368), and the lag in days for the maximum correlation of 0.4144 in mortality rate was found to be 171 days.

**Figure 3:**
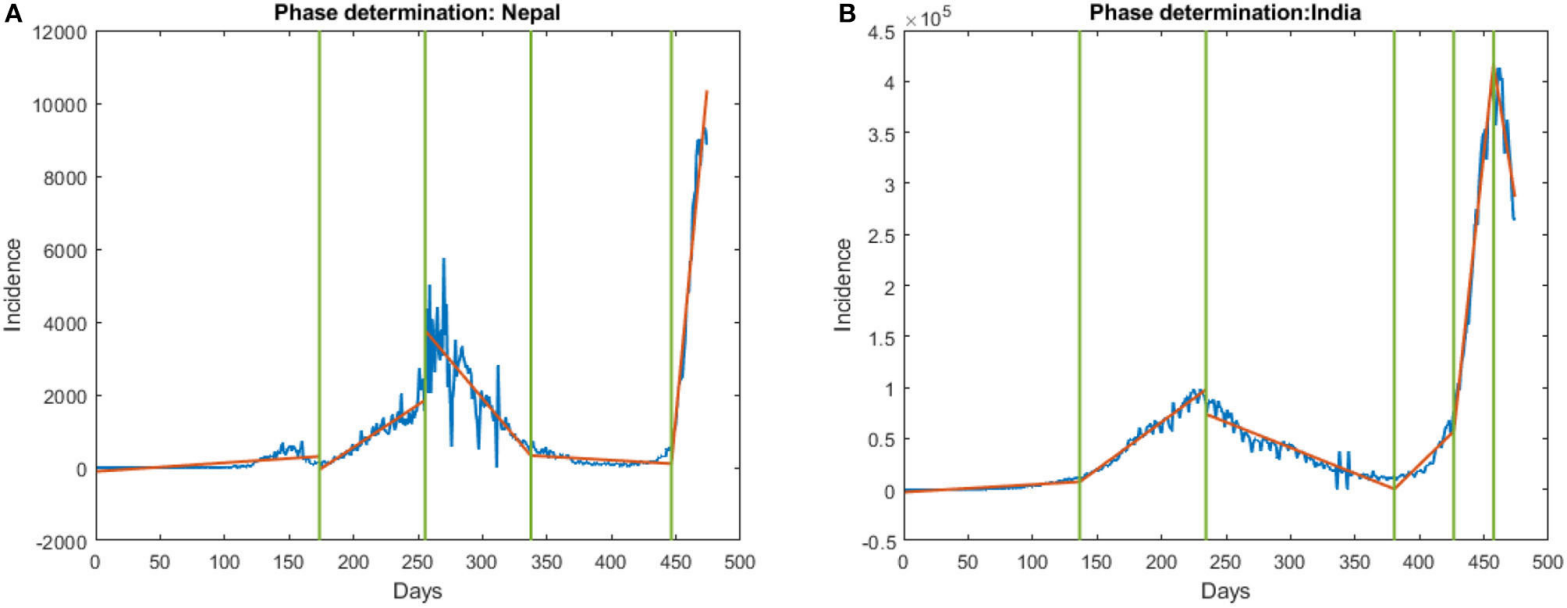
Onset and end of phase in **(A)** Nepal and **(B)** India. The first vertical line represents the onset of the first phase and the second represents the onset of the end of the first phase. Similarly, the third line indicates the end of the first phase.

We then studied the relationship in each COVID-19 wave separately. For which, we first detect the waves in the COVID-19 incident by observing the change in the statistical property of the data which indicates the different stages of the wave. To detect the change, we used the multiple change-point detections (CPD) (19) method which gave the start, inflection, duration, and end of each wave. Based on the multiple change-point detection methods, we observed the first wave in Nepal starts on July 16, 2020, and ends on December 27, 2020, (see Figure 3A). Similarly, the first phase in India starts on June 14, 2020, and ends on February 13, 2021, (see Figure 3B). We then computed the distance correlation between the two countries for the first wave and found it to be 0.8145 (*p* < 0.001). The lag in the first wave for daily new cases and daily new deaths was found to be 2 and 20 days respectively (see Figures 2C,D). Similarly, we applied CPD to detect the second wave and observed the second wave in Nepal started on April 15, 2021, and it started on March 31, 2021, in India. The distance correlation between the incidence was found to be 0.9685 (*p* < 0.001) for the second wave. The lag in the second wave for daily new cases and daily new deaths was found to be 0 and 0 days, respectively (see Figures 2E,F).

The reproducibility of the method was tested on other neighboring countries with open borders: the United Kingdom and Ireland, and Russia and Belarus. Between the UK and Ireland, the distance correlation for the daily new cases was found to be non-linearly dependent with a correlation coefficient of 0.568 (*p* < 0.01, *N* = 445), and the lag in days for the maximum correlation was found to be 23 days (see Supplementary Figure 1). The distance correlation for the daily new death was found to be non-linearly dependent with a correlation coefficient of 0.615 (*p* < 0.01, *N* = 434), and the lag in days for the maximum correlation was found to be 2 days (see Supplementary Figure 1). Similarly, between Russia and Belarus, the distance correlation for the daily new cases was found to be non-linearly dependent with a correlation coefficient of 0.794 (*p* < 0.01, *N* = 442), and the lag in days for the maximum correlation was found to be 8 days (see Supplementary Figure 2). The distance correlation for the daily new death was found to be non-linearly dependent with a correlation coefficient of 0.853 (*p* < 0.01, *N* = 414), and the lag in days for the maximum correlation was found to be 0 days (see Supplementary Figure 2).

## Discussion

For this study, we selected the two neighboring countries Nepal and India as both of them are fairly globalized (4) and share an open border. Our findings indicate that COVID-19 incidence in Nepal is significantly correlated with the incidence in India. It has been shown that the time to act is one of the most important measures for any health organization in curtailing the current COVID-19 pandemic (16, 23). While the timing of non-pharmaceutical interventions with respect to waves is fairly well-known, it is challenging to detect the wave itself and determine the optimal date prior to the start of the wave. The CPD can be used to detect the wave and the lag in days can be used to plan for pandemics in the future. The distance correlation in addition to cross-correlation can provide a way to estimate the timing to enact non-pharmaceutical measures. The high correlation observed in the second peak (Figure 2A) of incidence with a lag of 6 days can be attributed to the travelers crossing the open border between Nepal and India. It is not difficult to miss the detection of COVID-19 cases at the border since the incubation period of the virus is 1 to 14 (24) days and people might be tested as false negative during this period. The maximum correlation detected by our method is for the days within the incubation periods, which means most of the cases might not have been detected in the early days of infection and could have easily crossed the border. The border quarantine centers were not effective as a few people fled the centers (25). The undetected or the false-negative tests can even give people a false sense of security to travel between the countries and be the root of the spread of the virus. The lag in daily new cases in the first wave is 2 days (Figure 2C) whereas the lag in the second wave is 0 days (Figure 2E). The difference in lags between the first and second waves may be due to the reopening of the border before the start of the second wave (21, 26, 27), unlike the first wave when the border was closed and border quarantine was mandatory for all the returning workers of both countries (28).

Our finding sheds light on the correlation of daily new cases in two neighboring countries with an open border and how the lag in correlation can be leveraged to help the countries plan and apply non-pharmaceutical measures to reduce the spread of the virus. The cross-border travel restriction in the early days can help the countries keep the pandemic under control (4, 29, 30), as it has been shown that closing the border after the introduction of the virus has very little effect in preventing within-country spread (31, 32). The knowledge about future waves is essential for policymakers to make an informed decision on border closure in the early days as it is critically associated with the legal and diplomatic agreements and regulations between two countries. This study can provide a beacon signal of an imminent COVID-19 wave by estimating the time of resurgence. The knowledge of lag in days of COVID-19 incidence can act as a precursor to the government and public to be vigilant and practice safety measures, learning from Vietnam (33), such as restricting people from crossing the country, the establishment of effective screening methods at the border, and maintaining on-site healthy and safe isolation centers as no fully proven and specific antiviral treatment for the coronavirus exists (34). The stringent travel restriction between countries like Nepal-India with a large and multilayered socio-economic network can have a great impact on the pandemic dynamics (35). Nevertheless, the gain from the border closures can only be appreciated by combining it with other preventive measures like social distancing, intensive surveillance, testing, and contact tracing as demonstrated by Vietnam (33, 36). The strict travel restriction, at the very least, can delay the arrival of the virus and permit some time to prepare the health infrastructure (32, 37).

In contrast to the high correlation seen in the incidence, the correlation for daily new death is lower. The lower correlation in death can be accounted for by the unequal health facilities available in these countries. It is well-known that the unequal distribution of health infrastructure between two countries plays a significant role in the impact on casualties and disease transmission (29). The difference in the strength of health infrastructure (Table 1), testing facilities, and public health policy and poverty rate within Nepal and India may explain the observed lower correlation in the daily deaths count whereas the open border might be a contributing factor for high correlation in daily new cases. The lag in daily new death in the first wave is 20 days (Figure 2D) whereas the lag in the second wave is 0 days (Figure 2F) which may be due to the more lethal variant in the second wave, the lull in the travel restrictions (27), and the fact that border isolation centers were more effective in the first wave (40, 41).

**Table 1:** Health care/facilities indicator [source: World Bank (38, 39)].

**S.N**.	**Indicator**	**Nepal**	**India**
(1)	Number of doctors per 1,000	0.7	0.9
(2)	Hospital beds per 1,000	0.3	0.5
(3)	Current health expenditure per capita (current US$)	57.85	72.83
(4)	Nurses and midwives (per 1,000 people)	3.1	1.7
(5)	Poverty headcount ratio	25.2 (2010)	21.9 (2011)

The realization of containment, once the outbreak has occurred, depends on the adaptation time. The time window to apply containment intervention is very short and its efficiency decreases with the delay in the adaptation time (23). For the countries with a small population like Nepal, one of the efficient non-pharmaceutical measures to decrease COVID-19 spread is early isolation of asymptotic individuals, and the effectiveness of the strategy demands the realization of a huge number of daily new tests in the early days of the infection (16, 29). The time to relax the lockdown should be carefully be studied as it can have positive as well as negative impacts depending upon the country (42). Likewise, social policy and programs based on social determinants of health must be enacted by policymakers, stakeholders, and researchers to embrace all sectors of society.

To test the reproducibility of our method, we have extended the analysis to four other countries with an open border. The analysis for United Kingdom-Ireland and Russia-Belarus suggests that the delay in closing the border should not be more than 23 days between the UK and Ireland, and should not be more than 8 days between Russia and Belarus. The maritime boundary between the United Kingdom and Ireland might be a reason for the larger lag in incidence between these countries as compared to Nepal-India and Russia-Belarus.

Despite the promising results, the analysis considered here has few limitations. This analysis uses data that is different from the true data on the ground due to less testing and inefficient counting of new and death cases by Nepal and India (43–46). Similarly, multivariate distance correlation can be used to determine the dependencies by including other factors of transmission and vaccination rate. In future study, local micro-events like public gatherings, which can affect the results, should be taken into account while analyzing the outbreak. This work can further be extended by incorporating other pandemic outbreaks to generalize the analysis and findings.

## Conclusion

In this paper, we analyzed the Covid-19 incidence and deaths between countries with an open border to approximate the border closing data with respect to the beginning of waves seen in the neighboring country. We conclude that the non-linear correlation method, along with cross-correlation, can be applied to determine the dependencies and approximate the lead/lag in days of a Covid-19 wave between two neighboring countries with an open border. We tested the method on Nepal and India; one of the major findings of this analysis is that closing the border should not be delayed more than 6 days after detection of a Covid-19 wave in the neighboring country to stop/reduce the spillover.

## Data Availability Statement

Publicly available datasets were analyzed in this study. This data can be found here: https://github.com/owid/covid-19-data COVID-19. Data Repository by the Center for Systems Science and Engineering (CSSE) at Johns Hopkins University (JHU).

## Author Contributions

AS conceived and designed the concept and wrote the paper. AK and AS performed the literature review. AK contributed to analysis tools. Both authors have read and agreed to the published version of the manuscript.

## Conflict of Interest

The authors declare that the research was conducted in the absence of any commercial or financial relationships that could be construed as a potential conflict of interest.

## Publisher's Note

All claims expressed in this article are solely those of the authors and do not necessarily represent those of their affiliated organizations, or those of the publisher, the editors and the reviewers. Any product that may be evaluated in this article, or claim that may be made by its manufacturer, is not guaranteed or endorsed by the publisher.
