# Supplementary material for: Early Detection of COVID-19 Waves From Cases in a Neighboring Country With an Open Border
Source: Front Public Health. 2021 Oct 29;9:739738. doi: 10.3389/fpubh.2021.739738 (PMC8585937; doi:10.3389/fpubh.2021.739738)
Supplement: Supplementary file 1 [file Data_Sheet_1.docx]

**Supplementary materials**

| (A)  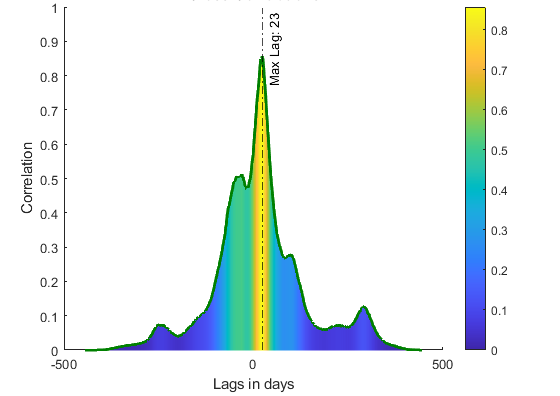 | (B)  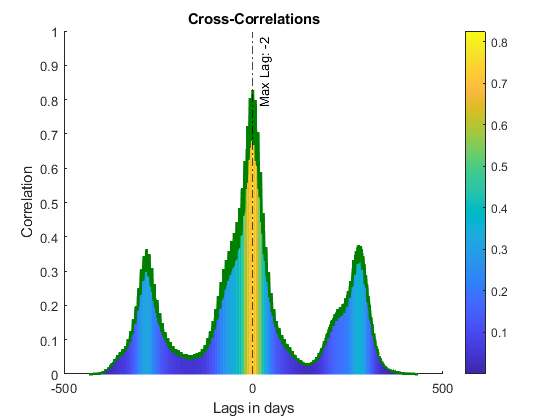 |
| --- | --- |
| (C)  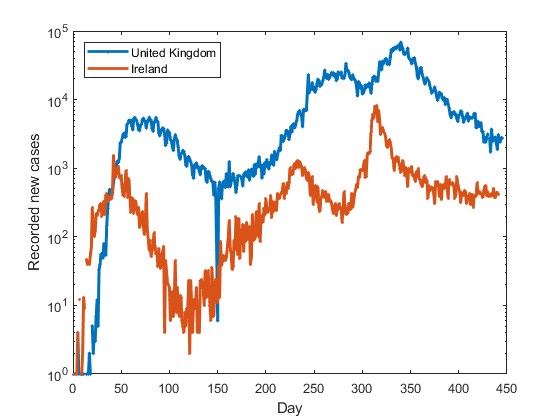 | (D)  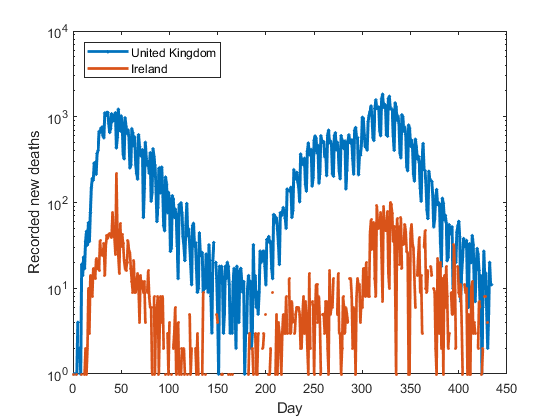 |
| Supplementary Figure 1: Cross-correlation between United Kingdom and Ireland with lags in days. The color represents the magnitude of cross-correlation at each lag. (A) Cross-Correlation of incidence new cases (B) Cross-Correlation of incidence new deaths (C) Recorded new cases of COVID-19 in United Kingdom and Ireland, (D) Recorded new deaths of COVID-19 in United Kingdom and Ireland. Note: the y-axis is in log scale to make the cases visible | |

| (A)  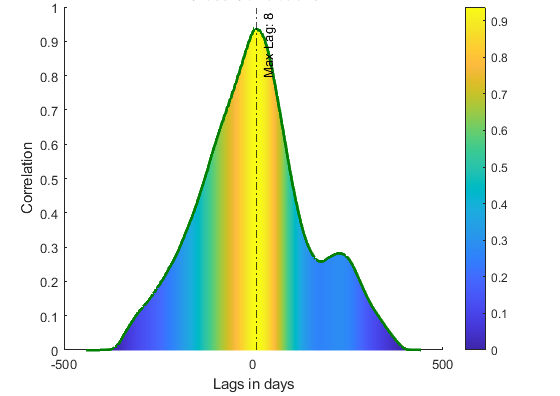 | (B)  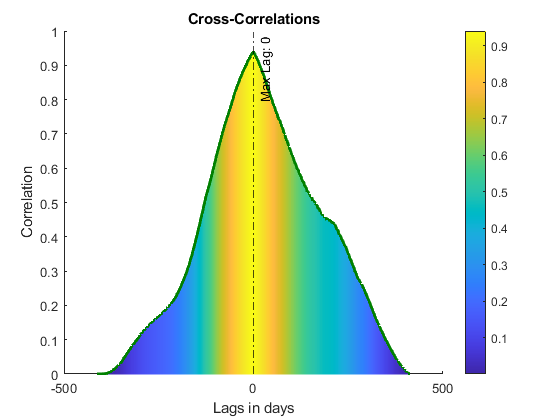 |
| --- | --- |
| (C)  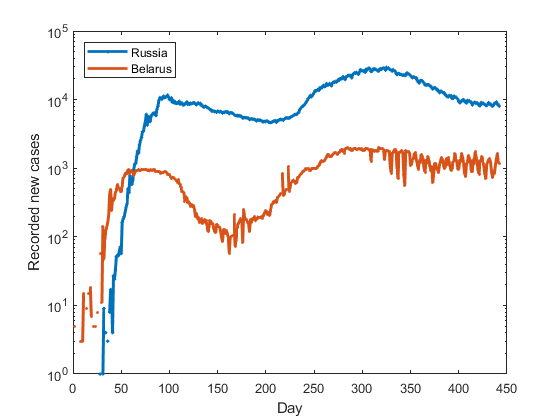 | (D)  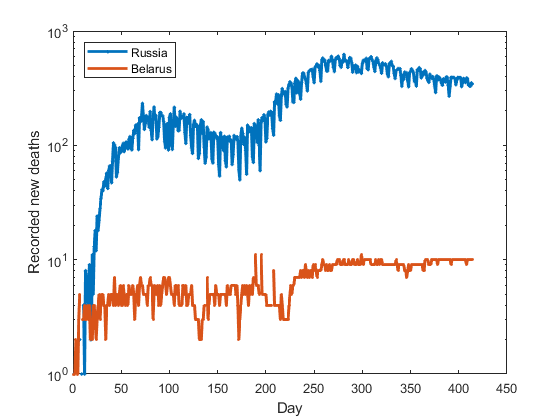 |
| Supplementary Figure 2: Cross-correlation between Russia and Belarus with lags in days. The color represents the magnitude of cross-correlation at each lag. (A) Cross-Correlation of incidence new cases (B) Cross-Correlation of incidence new deaths (C) Recorded new cases of COVID-19 in Russia and Belarus (D) Recorded new deaths of COVID-19 in Russia and Belarus. Note: the y-axis is in log scale to make the cases visible | |
